# Some Observations on Coccidia in the Renal Epithelium of the Mouse

**Published:** 1889-07

**Authors:** Theobald Smith

**Affiliations:** Department of Agriculture, Washington, D. C.


### ART. XIV.—SOME OBSERVATIONS ON COCCIDIA IN THE RENAL EPITHELIUM OF THE MOUSE.

BY THEOBALD SMITH, M.D.,
*Department of Agriculture, Washington, D. C.*

DURING October of last year, coccidia were discovered by the writer in the kidneys of three house-mice, inhabiting the epithelial cells of the convoluted tubules. The unusual situation of these intracellular parasites and the evident degeneration of the organ caused by them, were the inducements to a more careful study.

Unfortunately, other work prevented any thorough investigation at the time, and, the hope was entertained that more material would be forthcoming when needed. This hope has not been fulfilled up to the present time, and the few facts that could be collected are published now as a preliminary note to further investigations. The mice had been kept for an indefinite length of time, perhaps several months, in a cage about a foot and a half long and one foot wide. Subsequently they were inoculated with hog cholera bacilli, and their premature death within two or three days aroused suspicion, since mice live on an average from seven to ten days after inoculation. The kidneys presented an abnormally pale yellow appearance, and without doubt, the degenerated condition of these organs had greatly reduced the vitality of the mice and their resistance to the invasion of the pathogenic bacteria. It should be stated however that they appeared perfectly well at the time of inoculation.

From the kidneys of these mice, minute fragments of the cortex were teased and crushed in salt solution, in order to determine the form of the parasites in the fresh condition. In this way large numbers of nearly spherical bodies were brought into view, of which the longer diameter measured about 16.2 $\mu$, the shorter 12.6 $\mu$. Each body consisted of a cyst the interior of which was densely packed with crescent-shaped bodies. There could be no doubt that this parasite belonged to the coccidia, a family under the general group of sporozoa, and that the stage represented by the presence of the crescent-shaped or falciform bodies was the final or spore state in the life of the micro-parasite.[1]

[1]Coccidia are one of five groups of parasitic protozoa brought together by Balbiani, (Lecons sur les sporozoaries, 1884.) under the name sporozoa. They are 1. the *gregarinidæ* found almost exclusively in invertebrates, 2. the *coccidiidæ* chiefly parasites of vertebrates, 3 the *myxosporidiæ* of fishes, and 4. the *sarcosporidiæ* known also as *psorospermia* in the muscular tissue of many domesticated mammals. To these four divisions he has added a fifth, the *microsporidiæ* of insects, a member of which causes the pébrine disease of silk-worms.

Coccidia are distinguished by their intracellular mode of existence. They live in the protoplasm of epithelial cells and have thns far been encountered in the epithelium of the intestines, the liver and the kidneys. Recently Steinhaus (Archiv. f. path, Anat. etc. cxiv. 1889, 176) described a coccidium which lives in the nucleus of the intestinal epithelium of *Salamandra maculosa*. This is the first instance of intra-nuclear parasitism on record. They begin their life cycle as amœbiform bodies within the protoplasm (or nucleus) of epithelial cells. Here they enlarge and in some species leave the cells and enter upon the spore state outside the host. In other species (and in the one under consideration) they complete their life cycle as parasites. This is accomplished by the formation of one or more spores within a cyst into which the amœbiform body is transformed. The spore breaks up into a variable number of bodies having the shape of crescents and known as the falciform bodies. Under suitable conditions the crescents are transformed into amœbæ to take up anew the life cycle. The ravages of coccidia are seen most readily in the liver of rabbits. Epizootics of liver disease carrying off large numbers of rabbits are caused by *coccidium oviforme.* The writer has seen the liver so thoroughly infested with coccidia cysts that the organ as a whole appeared from the exterior like a thin yellowish bag filled with peas. Scarcely any of the original tissue was to be seen.

The cyst-wall was represented optically by a narrow, sharply defined dark line, and when crushed under the cover-glass in order to force out the contents its shrunken remains appeared transparent and colorless. The cysts contained as nearly as could be ascertained, about twenty crescent-shaped bodies filling out the entire cavity. In form these crescent-shaped, or more properly, falciform bodies might be compared to bananas, having perhaps a little greater curve than the average fruit of this name. Their two extremities were blunt and rounded. Their length was 7μ, their greatest diameter 1.8μ. The substance composing them appeared homogeneous in the fresh condition. When packed away in the cyst, their long axes were in general parallel so that their extremities came to lie at two opposite poles. When the axis connecting the poles was at right angles to the optical plane, or vertical, and the equatorial plane was focused, upon the cyst appeared filled with small circles representing the falciform bodies in optical section. When the axis was inclined, the effect of a turbine was produced as the objective was slowly brought down.

In the teased preparations these spore cysts were very numerous so that every field of the microscope contained from ten to fifteen or more, in part isolated, in part united into groups consisting of from several to twelve each, and surrounded by a very delicate line which in some cases could be demonstrated as the attenuated periphery of the cell body. These groups, in other words, were contained in large vacuoles formed in the protoplasm of the epithelial cells between the free border of the cells and their nuclei. None of the cells observed in the teased preparations contained more than one vacuole. The wall of the vacuole either adhered snugly to the group of cysts thus forming a rather irregular, elongated sac, or the vacuole was spherical and but partly filled up by these cysts. One can imagine the size of such a cell containing about ten ripe coccidia. In the same specimen other bodies were observed in a similar situation most likely earlier stages in the life history of the coccidia. As sketched at the time two forms presented themselves, also appearing in groups, each in a large vacuole of an epithelial cell. The presumably younger stage consisted of a spherical vesicle about one half the size of the ripe coccidium, its cell wall a delicate, homogeneous sac. In the transparent colorless contents was imbedded a single refracting body or nucleus somewhat eccentrically situated. The second forms also found in groups inhabiting vacuoles in the cells were intermediate in size between the forms just described and the ripe ones. The sac was indicated by a rather faint line, and was entirely filled by coarse, refracting granules. The relation which these forms bear to one another as well as the intimate structure of each, will require more careful examination. The unique position which they occupy in organs so isolated from the outside world as the kidneys should enable us to get at those phases of their life history through which they pass in the body of the mouse, more easily than if they were parasites in the epithelium of the digestive tract, which is constantly in contact with so much miscellaneous matter.

To determine the relation of the coccidia to the kidney structures and to their distribution, these organs were hardened in Müller's fluid and alcohol, imbedded in paraffine, and cut dry in series.[1] Sections stained in hæmatoxylin and cleared in clove oil fluorescine were most satisfactory. It required, however, but very little observation to discover that nothing could be learned of the morphology of the coccidia themselves from hardened material. They were limited to the cortex where they inhabited the convoluted tubules. These were considerably dilated and the epithelium almost completely destroyed. The ripe cysts were easily distinguished. Some remained unstained and the falciform bodies could be fairly well outlined, the whole cyst having a rather brilliant lustre. Other cysts in which the falciform bodies were present, took the stain. The difference in this regard may be due to the more mature stage, and hence to a denser, less pervious envelope of the unstained forms. The ripe cysts usually appeared in large numbers in the tubes, loosely filling them up. In many tubes the very delicate sac surrounding certain groups could also be made out, and the latter identified as projections or bulgings of the epithelial layer. In a certain number of tubes densely packed clusters of what were probably the younger stages referred to, could be seen enclosed in a sac and directly adherent to the epithelium.

---

[1] In order that the contents of the tubules should not be lost during the staining they were fastened to cover-glasses. One or two drops of 70 per cent. alcohol were placed on the section and permitted to evaporate in a thermostat at 37°C for from two to twenty-four hours. The sections thereby became firmly attached, so that the subsequent removal of the paraffine and the staining did not wash out the coccidia. The writer has found that the device of fastening the sections to cover-glasses makes subsequent manipulations much simpler than when they are attached to slides. For the method (Gaule's) I am indebted to Prof. Welch's paper on the structure of white thrombi. *Trans. Path. Soc. Philadelphia, XIII, 1887.*

The hardening process had made the outlines and forms of the younger cysts quite unrecognizable. The distension of the cortical tubes reached its climax in one which, in the hardened condition, was not less than 70 $\mu$ in diameter and a single very thin cross section contained at least eight clusters of immature and ripe coccidia. It is impossible to give more than a very rough estimate of the number of parasites present in the kidney. Allowing .02 mm. for the maximum thickness of each transverse section, containing not less than 200 cysts we would have in a kidney one cm. long not less than 100,000 individuals.

Owing to the scarcity of the material on hand only one effort was made to induce the disease in mice through the food, but with negative results. A kidney of one the mice, containing ripe coccidia had been kept in a refrigerator in normal salt solution for nearly a month. Many of the parasites appeared unchanged at the end of this time. One-half of the kidney was thoroughly broken up and mixed with some bread and placed in an anatomical jar containing two mice. Owing to the infection, of the organ thus fed, with hog cholera bacilli, the mice became very sick but did not die, very probably because the bacilli had become attenuated meanwhile. One mouse was killed thirty-three days after feeding. No coccidia were found in the kidneys. There was, however, a large abscess in the liver, due to the hog cholera bacilli, the abscess resulting from the breaking down of tissue which had undergone coagulation-necrosis. The second mouse was killed fifty-one days after feeding. In the kidneys no traces of coccidia were found. The former presence of hog cholera bacilli was manifested by several abscesses, one extending from the papilla as a broadening wedge to the cortex. This single experiment can have no weight in deciding upon the direct transmissibility of coccidia since the material was neither fresh nor pure. A considerable number of mice examined subsequently by teasing bits of cortex of the kidneys in salt solution appeared free from coccidia. This was not entirely true, however, as the following illustration shows. Portions of the kidney of one adult mouse were examined in this way and found free from coccidia. Both kidneys were then hardened and sections made to be compared with sections of the diseased kidneys. On examination a few tubules were found occluded with immature coccidia in groups of five or six. A few cysts containing falciform bodies were found soon after in the kidneys of another adult mouse.

The somewhat meager observations on this parasite would lead us to assume that the ripe cysts are discharged by way of the collecting tubules of the kidney and finally leave the body in the urine. The falciform bodies taken up with the food by other mice, are transformed into amœbæ which most likely reach the kidneys by way of the blood vessels. There they enter the epithelial cells of the cortex and finally undergo segmentation into the falciform bodies to be again discharged. The size of the vacuole will vary with the number of individuals which have penetrated into the same epithelial cell. How far the liver is implicated with the kidneys it is impossible to say, since microscopic sections as well as fresh teased preparations in one case gave no positive information. It must however be borne in mind that earlier stages of the parasite may have been overlooked. The intestines of the diseased mice were not examined and this is the more to be regretted in view of Eimer's investigations summarized below. The intestines of several apparently healthy mice were subsquently examined but no coccidia discovered.

The mode of exit and entrance of the parasites, given above, assumes that there is no state outside the host necessary to make them infectious, *i. e.*, they mature in the host and pass directly from one animal to another. In fact, the same animal may be constantly re-infecting itself. This theory gains strength from two considerations: first, the very extensive infection of the organs examined and the different stages represented in the same organ; second, the presence of a few coccidia in apparently healthy mice. A long period of confinement in a small cage, as was the case with the mice examined, may expose them to continuous infection so that a single mouse with a few coccidia to begin with may after a time cause an extensive invasion of coccidia in itself and others confined with it. These as well as other problems the writer hopes to attack again ere long and simply presents the theories as a means of knitting together the facts actually observed.

The species under consideration has not yet been described. In 1870 Eimer[1] found in the intestines of three mice coccidia, many of them within the protoplasm of the epithelial cells. They are

---

[1] Ueber d. segenannten ei-oder kugelförmigen Psorospermien d. Wirbelthiere. Würzburg, 1870.

described as round or oval. The round forms had a diameter of 18 μ, the two axes of the oval ones were 16 μ and 26 μ respectively. The falciform bodies varied in length from 9.25 μ to 16.3 μ and contained in their interior three or four brilliant granules. Owing to the large number of these free crescents in the intestines Eimer was able to observe their movements on the warm stage. These consisted in bending and straightening. When bent they resembled the letter C with its free ends near each other. Sometimes when only one extremity would bend they assumed a form like the figure 6. They subsequently became transformed into spheres with granules in their interior. These were found, from one to three in an epithelial cell, where they enlarged, became encysted and developed in their interior eight falciform bodies. They were not found in other organs.

A comparison of the kidney coccidium with this species, especially with reference to the size of the cyst, the size and number of the falciform bodies, and the situation, will show at once that they are different species, most likely of the same genus *Eimeria*, the intestinal coccidium being known as *E. falciformis*. The presence of coccidia in the kidneys of other animals may not be so rare an occurrence after all. A. Pachinger[1] briefly mentions the discovery of coccidia in the more or less degenerated kidneys of three horses which he does not hesitate to consider identical with *Eimeria falciformis*. Coccidia have been encountered in the intestines of many domesticated animals including the cat, dog, rabbit and sheep and in man. In a case reported by Gubler[1] in 1858, a fatal liver disease was produced by coccidia. It is not improbable that now and then infection with these micro-parasites occurs in man, which is overlooked because of the smallness of the objects and the ease with which they may be confounded with normal cell structures. The importance which parasitic protozoa are beginning to assume owing to their etiological relation to malarial fevers and their possible relation to some of the still obscure infectious local and general diseases should induce us to pay them more careful attention than they have hitherto received.

---

[1] Zool. Anzeiger. IX, 471. [2] Balbiani, *loc. cit.*, p. 95.

